# LSML-SF: a lightweight stacked ML approach for spreading factor allocation in mobile IoT LoRaWAN networks

**DOI:** 10.3389/frai.2026.1704369

**Published:** 2026-02-06

**Authors:** Arshad Farhad, Muhammad Ali Lodhi, Farhan Nisar, Hassan Jalil Hadi, Naveed Ahmad, Mohamad Ladan

**Affiliations:** 1 Department of Computer Science, Bahria University, Islamabad, Pakistan; 2 School of Information Engineering, Yangzhou University, Yangzhou, China; 3 Department of Physical and Numerical Science Qurtaba University & IT, Peshawar, Pakistan; 4 Center of Excellence in Cyber Security, Prince Sultan University, Riyadh, Saudi Arabia; 5 Software Engineering Department, Prince Sultan University, Riyadh, Saudi Arabia; 6 College of Computer and Information Sciences, Prince Sultan University, Riyadh, Saudi Arabia

**Keywords:** Internet of Things (IoT), LoRaWAN, machine learning, resource allocation, spreading factor, stacked generalization

## Abstract

The expansion of the Internet of Things (IoT) into consumer applications demands robust and energy-efficient communication protocols. Long-range wide area network (LoRaWAN) is a key enabler, but its performance depends on optimal spreading factor (SF) allocation, where traditional adaptive data rate (ADR) mechanisms are inadequate in dynamic environments. This study presents a novel lightweight stacked-ML approach for spreading factor (LSML-SF) allocation in mobile IoT LoRaWAN network. We propose a stacked ensemble model that jointly combines a linear stochastic gradient descent classifier (log-loss), a gradient boosting model, and a deep neural network (DNN) through a logistic regression meta-learner. The LSML-SF is trained on a vast dataset of 225,109 samples generated from ns-3 simulations, and our model achieves an out-of-fold cross-validation accuracy of 85%. Importantly, we demonstrate the practical feasibility of our approach through a rigorous computational analysis, showing the DNN component requires only 12,602 parameters and 12.3k MAC operations per inference. When integrated into ns-3 simulations, our LSML-SF framework significantly outperforms traditional ADR mechanisms and existing ML approaches, improving the packet success ratio and reducing energy consumption, thereby extending the operational lifespan of consumer IoT devices.

## 1 Introduction

The vision of a seamlessly connected world is rapidly materializing through the Internet of Things (IoT), which has integrated into countless consumer domains. From smart agriculture and logistics to personalized healthcare and smart city infrastructure, IoT devices are revolutionizing how consumers interact with their environment (Almuhaya et al., 2022). A critical enabler of this revolution is low-power wide-area network (LPWAN) technology, which provides the essential connectivity for billions of devices. Among various LPWAN solutions, Long-Range Wide Area Network (LoRaWAN) has gained significant attention. This is due to its compelling trade-off between communication range, power consumption, and device cost (Farhad et al., 2020; Butt et al., 2025). Consequently, LoRaWAN is exceptionally suitable for consumer electronics applications.

**Figure 1:**
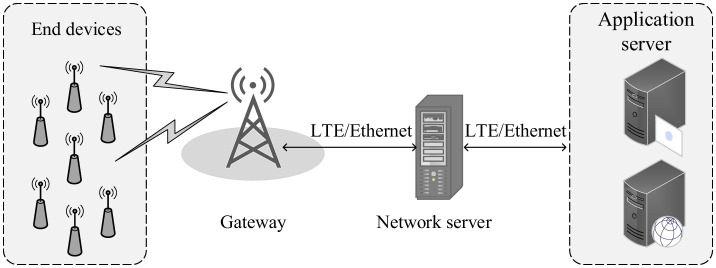
LoRaWAN network architecture illustrating the end-to-end communication flow. End devices transmit uplink packets to a gateway using spreading factors, while downlink messages and acknowledgments are relayed through the network server. The gateway forwards traffic to the network server over LTE or Ethernet, and application-level data are handled by the application server.

Figure 1 shows the basic architecture of a LoRaWAN network. End devices (EDs) transmit uplink data to a gateway (GW) using LoRa modulation, with spreading factors (SF) typically ranging from SF7 to SF12. The choice of SF directly affects communication behavior: lower SF values, such as SF7, provide higher data rates over shorter distances, whereas higher SF values, such as SF12, increase communication range at the cost of longer airtime and reduced throughput. LoRaWAN operates in a star topology, where EDs access the medium using an ALOHA-based uplink transmission scheme. After each uplink transmission, two receive windows are opened to enable downlink communication from the network server (NS) via the GW. The first window (RX1) opens 1 s after the uplink using the same channel and SF, while the second window (RX2) opens after 2 s on a predefined channel with SF12. These receive windows allow the NS to deliver acknowledgments or control messages when required. The GW connects to the NS through LTE or Ethernet backhaul links, which subsequently relay processed data to the application server for advanced processing. This structure supports robust, low-power connectivity for extensive IoT deployments, as detailed in recent surveys on LoRaWAN scalability (Jouhari et al., 2023), machine learning enhancements (Farhad and Pyun, 2023b), security vulnerabilities (Hessel et al., 2022), and adaptive data rate (ADR) optimizations (Lehong et al., 2024).

Within the LoRaWAN architecture (as illustrated in Figure 1), the NS implements the ADR mechanism, which dynamically adjusts the SF and transmission power (TP) based on the signal-to-noise ratio (SNR) history of the last 20 packets received from each ED. This adjustment aims to optimize network performance by selecting an appropriate SF and TP configuration that balances range, data rate, and energy consumption, though its efficacy is challenged by the dynamic nature of real-world consumer environments. However, the dynamic nature of real-world consumer environments, characterized by device mobility, signal fading, and interference, poses a significant challenge to traditional ADR. Its slow reaction time and inability to model complex channel dynamics often lead to suboptimal SF assignments, resulting in packet loss, increased network congestion, and accelerated battery drain (Rehman et al., 2025; Ullah et al., 2025). This directly degrades the performance and user experience of consumer IoT products.

Machine learning (ML) offers a powerful paradigm to overcome these limitations by learning complex patterns from data for optimal SF allocation. Recent works have explored models like deep neural networks (DNNs) (Farhad and Pyun, 2023a) and gradient boosting (Minhaj et al., 2023). However, a significant gap remains in developing a solution that is not only highly accurate but also demonstrably feasible for deployment on the computationally constrained microcontrollers that are ubiquitous in consumer EDs. Many high-accuracy models are too complex for practical implementation, while simpler models may lack the required predictive performance. This study addresses this gap by introducing a high-performance, yet deployable, stacked ensemble ML framework for SF classification. Our approach does not rely on a single model but leverages the complementary strengths of multiple learners to achieve superior and robust accuracy. Specifically, we present the lightweight stacked-ML approach for SF (LSML-SF) allocation in mobile IoT LoRaWAN networks. The proposed LSML-SF combines a linear SGD classifier (functioning as a support vector machine), a gradient boosting (XGBoost) model, and a DNN through a logistic regression meta-learner.

### 1.1 Contributions of the paper

The key contributions of the proposed LSML-SF are as follows:

1. We propose and implement a sophisticated stacked generalization pipeline that combines three diverse base learners: a linear SGD classifier, an XGBoost model, and a DNN. A logistic regression meta-learner is trained to optimally blend the predictions from these base models. This architecture is specifically designed to capture the complex, nonlinear relationships between device state, channel conditions, and the optimal SF, achieving an overall classification accuracy of approximately 88% across all six SF classes.
2. We develop a robust feature engineering strategy that expands a set of 5 base features (e.g., device location, distance, received power, SNR) into a rich set of 29 features. This includes rolling statistics (mean, std, min, max) to capture temporal dynamics and domain-informed interaction terms (e.g., distance × SNR) and nonlinear transformations (e.g., log(1+Distance)). This process provides the model with a highly informative input representation that is critical for achieving high accuracy.
3. We validate the effectiveness of our LSML-SF framework by integrating the pre-trained model into the ns-3 network simulator. Performance evaluation under mobile scenarios shows that our LSML-SF approach consistently and significantly outperforms traditional ADR mechanisms as well as other ML-based benchmarks, achieving improvements in packet success ratio (PSR) and reducing overall network energy consumption.

### 1.2 Structure of the paper

The rest of the study is organized as follows: Section 2 reviews existing ML approaches for LoRaWAN parameter optimization and identifies key research gaps. Section 3 introduces the proposed LSML-SF framework, covering dataset generation, feature engineering, model architecture, and training strategy. Section 4 evaluates the offline predictive performance of the stacked ensemble, including confusion matrix analysis, convergence, and deployment feasibility. Section 5 reports online ns-3 simulation results, highlighting improvements in packet success ratio, energy consumption, and packet loss ratios under mobility. Section 6 outlines current study limitations and future directions, while Section 7 concludes with key findings and implications for real-world IoT deployment.

## 2 Literature review

This section surveys recent ML approaches for optimizing communication parameters in LoRa and LoRaWAN networks, including SF, TP, bandwidth (BW), and coding rate (CR). The primary goal of these methods is to enhance overall network efficiency and performance. A comparative summary of these approaches is presented in Table 1.

**Table 1:** Summary of representative machine learning-based approaches for parameter optimization in LoRa and LoRaWAN networks, highlighting the targeted optimization problems, employed learning techniques, key reported performance gains, and the main limitations identified in each study.

**References and Year**	**Optimization problem**	**Methodology**	**Key results**	**Identified limitations**
Azizi et al. (2022)—2022	SF classification	Multi-armed Bandit (RL)	Improved PDR and energy efficiency	Computationally costly for EDs; requires online learning
Chen et al. (2023)—2023	SF classification	Markov Decision Process (MDP)	24–27% Reduction in energy consumption	Evaluated only in a simulated environment
Bertocco et al. (2023)—2023	Soil water volume estimation	Multi-sensor ML fusion	Lowest RMSE (1.53% with hybrid method)	Requires large, costly datasets for training
Vangelista et al. (2023)—2023	Device mobility classification	Support Vector Machine (SVM)	Accurate binary mobility classification	Integration with a subsequent ADR strategy
Farhad et al. (2022b)—2023	SF classification	Gated Recurrent Unit (GRU)	96% Accuracy; 98% PSR for 100 EDs	High computational complexity
Farhad and Pyun (2023a)—2023	SF classification	Deep Neural Network (DNN)	82% Accuracy; Improved PDR and energy	On-device inference increases energy consumption
Prakash (2025)—2025	SF Prediction via feature selection	Supervised ML (k-NN, DTC, RF, MLR)	Optimal RSSI + SNR features; High accuracy/F1	Limited to static feature sets; no real-time adaptation
González-Palacio et al. (2023)—2023	Path loss/shadowing for energy efficiency	Supervised ML (MLR, SVR, RF, ANN)	Up to 43% energy savings; RMSE 1.566 dB	Dependent on environmental sensor data availability
Hazarika and Choudhury (2024)–2024	SF allocation for mobile/static EDs	Hybrid (K-means + RL)	Improved PSR, energy, convergence time	Simulation-based; computational overhead at GWs

In Azizi et al. (2022), the authors investigated dynamic SF allocation in LoRaWAN using a reinforcement learning approach referred to as MIX-MAB. Their implementation relied on the LoRa-MAB Python simulator and focused on a single-gateway deployment with 100 static end devices uniformly distributed within a 4.5 km radius. The study followed the EU-868 MHz duty-cycle restriction of 1% and assumed a traffic profile of 15 uplink packets per hour with payload sizes of 50 B. To simplify the analysis, the evaluation was conducted under idealized conditions without ACK collisions. Within this controlled setting, the proposed approach achieved higher packet delivery ratios and improved energy efficiency when compared with existing RL-based baselines.

Building on reinforcement learning for parameter adaptation, the work in Chen et al. (2023) introduced the score table-based evaluation and parameter surfing (STEP) algorithm. The evaluation was carried out using the MULANE simulator in MATLAB, where STEP was benchmarked against standard ADR, Blind ADR (BADR), and LoRa-MAB. The results showed a noticeable reduction in energy consumption, ranging from 24% to 27%, highlighting the potential of table-driven learning strategies for SF optimization.

A different application of ML in LoRaWAN was explored in Bertocco et al. (2023), where the authors targeted underground monitoring scenarios. Their study used a laboratory-generated dataset collected from a sand-filled environment with varying soil moisture levels. Received Signal Strength Indicator (RSSI) measurements and moisture sensor readings were employed to train and evaluate several ML-based estimation strategies. Specifically, the authors compared sensor calibration using ML, virtual sensing based solely on RSSI, and a hybrid approach combining physical sensor data with RSSI information. Among these, the hybrid method achieved the lowest estimation error, with an RMSE of 1.53%, outperforming both the sensor-only and RSSI-only approaches.

A gated recurrent unit (GRU)-based solution for efficient resource management, specifically SF allocation, was proposed in Farhad et al. (2022b) to improve the packet success ratio (PSR) of LoRaWAN networks. The dataset for this study was generated using the ns-3 simulator and included 500 static EDs. It comprised four key features: X-coordinate, Y-coordinate, signal-to-noise Ratio (SNR), and received power. The GRU model architecture consisted of two layers and one fully connected layer, followed by a softmax activation function for classification. This model achieved a classification accuracy of 96%. The weights and biases from the best-performing model were saved and later integrated into the ns-3 simulator for dynamic SF allocation during network simulations. As a result, the proposed GRU method achieved a high PSR of 98% for a network of 100 static EDs and 73% for a larger network of 600 EDs.

Expanding on this work, the authors in Farhad and Pyun (2023a) employed a DNN model tailored for both static and mobile LoRaWAN networks, utilizing the same dataset as in Farhad et al. (2022b). The dataset was partitioned into groups based on successful ACK reception. Each group was labeled with its single most efficient SF, and this processed data was used to train the DNN. Their model comprised five fully connected layers with varying numbers of neurons and a final softmax layer for SF classification, achieving an accuracy of 82%. When this pre-trained model was deployed within the ns-3 simulator for live network simulations, it demonstrated superior performance in PDR, energy consumption, and convergence period compared to traditional methods like ADR and other ML approaches like SVM. Consequently, the DNN-based approach outperformed GRU, LSTM, and SVM models.

In Prakash (2025), the authors addressed SF prediction in large mobile LoRaWAN-based IoT networks through effective feature selection. Using a publicly available dataset with over 930,000 datapoints, they evaluated k-nearest neighbors (k-NN), decision tree classifier (DTC), random forest (RF), and multinomial logistic regression (MLR) across 31 feature combinations from key parameters like RSSI, SNR, frequency, distance, and antenna height. The RSSI and SNR combination emerged as optimal, achieving high accuracy and F1 scores. This work highlights reduced dataset collection costs and extended battery life for LoRaWAN devices.

The authors in González-Palacio et al. (2023) proposed ML-based models for combined path loss and shadowing in LoRaWAN to enhance energy efficiency. Incorporating environmental variables such as temperature, relative humidity, barometric pressure, particulate matter, and SNR, they fitted models using multiple linear regression (MLR), support vector regression (SVR), random forests (RF), and artificial neural networks (ANN). Achieving RMSE up to 1.566 dB and *R*^2^ up to 0.94, their approach improved the ADR algorithm, reducing link margin and saving up to 43% energy compared to traditional ADR.

For hybrid techniques, Hazarika and Choudhury (2024) introduced the intelligent spreading factor allocation (iSFA) approach for mobile and static EDs in LoRa-based networks. Combining K-means clustering at EDs (based on features like unique ED ID, SF, SNR, RSSI, energy ratio, and packet success) with RL at GWd (optimizing DR, TP, and latency), iSFA reduced packet loss, convergence time, and energy consumption while improving throughput and PSR in simulations.

While the existing literature demonstrates significant progress in optimizing LoRaWAN parameters through ML, three critical research gaps remain unaddressed: (1) *Interpretability*—Prior works (Farhad et al., 2022b; Farhad and Pyun, 2023a; Azizi et al., 2022) focus predominantly on performance metrics without providing explanations for the models' decisions, which limits trust and hinders operational deployment in critical applications; (2) *Mobility Handling*—Most solutions (Chen et al., 2023; Vangelista et al., 2023) either ignore dynamic environments entirely or treat mobility classification and resource adaptation as separate, disconnected processes; and (3) *Energy-Accuracy Trade-off* —Current methods (Bertocco et al., 2023) can achieve high accuracy but often at prohibitively high computational costs for resource-constrained EDs. While this work directly addresses (2) *mobility handling* and (3) *energy–accuracy trade-off* through a mobility-aware stacked learning framework and lightweight inference design, (1) *model interpretability* remains an open challenge for ensemble and deep learning-based ADR solutions, including the proposed LSML-SF. Accordingly, this study focuses on improving reliability and energy efficiency under mobility, while acknowledging interpretability as an important direction for future research in ML-driven LoRaWAN resource allocation.

## 3 Proposed LSML-SF framework

**Figure 2:**
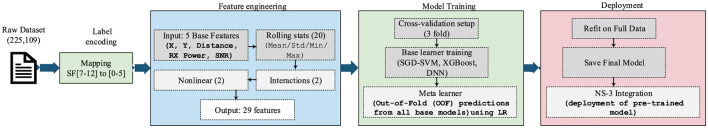
Proposed LSML-SF workflow illustrating the offline training and online deployment stages. The pipeline includes dataset generation, feature engineering, training of heterogeneous base learners, stacked ensemble learning using out-of-fold predictions, and integration of the trained model within the ns-3 simulation for adaptive SF selection.

This section describes the proposed LSML-SF framework for SF classification in LoRaWAN networks. The framework is designed to balance predictive accuracy with computational efficiency, making it suitable for deployment in resource-constrained EDs. As illustrated in Figure 2, the overall pipeline consists of dataset preparation, feature engineering, ensemble model training, and online deployment within the ns-3 simulator.

### 3.1 Dataset description and pre-processing

The dataset used for training and validation was generated using a widely adopted LoRaWAN network simulator^1^ (Magrin et al., 2019, 2017; Farhad et al., 2019). This choice ensures consistency with prior LoRaWAN studies and preserves realistic physical-layer behavior. A representative subset of the dataset is shown in Table 2.

**Table 2:** Representative sample of the raw LoRaWAN dataset used for model training and validation, showing packet-level attributes including device identifier, acknowledgment status, spatial location, link-quality indicators, and the corresponding SF.

**ED**	**Group**	**ACK**	**X (m)**	**Y (m)**	**Distance (m)**	***P*_*rx*_ (dBm)**	**SNR (dB)**	**SF**
110	1	0	4260.97	–996.186	4375.89	–132.872	–15.8407	7
375	1	1	2598.60	–3465.96	4331.95	–129.237	–12.2063	7
152	1	0	–1074.27	–4285.88	4418.48	–130.064	–13.0330	7

The simulation scenario consists of 500 static EDs uniformly distributed within a circular area of radius 5 km, with a single gateway (GW) positioned at the center. For each ED location, six packet transmission attempts are simulated using spreading factors ranging from SF7 to SF12. Each observation corresponds to a single transmission attempt and includes the ED identifier, packet group index, acknowledgment (ACK) status, spatial coordinates (*X*, *Y*), distance to the GW (*d*), received signal power (*P*_*rx*_), signal-to-noise ratio (SNR), and the SF used for transmission.

The target variable for supervised learning is the optimal spreading factor, denoted as SF^*^. For a given ED *i* with *n* transmission attempts, SF^*^ is defined as the lowest SF that results in a successful ACK. This definition reflects the operational objective of maintaining reliable communication while minimizing airtime and energy consumption. The labeling rule is formalized in Equation 1.


$$SF_{i}^{*}=\{\begin{array}{ll} \min\{SF_{i,j}|ACK_{i,j}=1\}, & \text{if }\exists j\text{ such that }ACK_{i,j}=1,\\ SF_{12}, & \text{otherwise.} \end{array}$$ (1)

The categorical SF labels are encoded as integer class indices *y*∈{0, 1, …, 5} corresponding to {*SF*7, …, *SF*12} using a LabelEncoder. To address class imbalance, class weights are introduced, with additional emphasis on higher SFs due to their disproportionate impact on network capacity and energy consumption. The class weight *w*_*c*_ is computed as


$$\begin{array}{c} w_{c}=\alpha_{c}\cdot \frac{N}{N_{c}\cdot C}, \end{array}$$ (2)

where *N* is the total number of samples, *N*_*c*_ is the number of samples in class *c*, *C* is the number of classes, and α_*c*_ is a class-specific scaling factor (α_*SF*11_ = 1.6, α_*SF*12_ = 1.8).

### 3.2 Feature engineering

To improve predictive performance beyond raw measurements, a total of 29 engineered features are constructed. The base feature set $F_{\text{base}}=\left\{\text{X},\text{Y},\text{Distance},\text{RX Power},\text{SNR}\right\}$ is augmented using temporal statistics and nonlinear transformations.

For each base feature $f\in F_{\text{base}}$, rolling statistics with window size *W* = 5 are computed on a per-ED basis to capture short-term temporal variation:


$$\begin{array}{c} f_{\text{mean}}=\text{rolling mean}\left(f,W\right) \end{array}$$ (3)

$$\begin{array}{c} f_{\text{std}}=\text{rolling std}\left(f,W,\text{ddof}=0\right) \end{array}$$ (4)

$$\begin{array}{c} f_{\text{min}}=\text{rolling min}\left(f,W\right) \end{array}$$ (5)

$$\begin{array}{c} f_{\text{max}}=\text{rolling max}\left(f,W\right) \end{array}$$ (6)

In addition, domain-informed interaction terms and nonlinear transformations are included to better represent channel behavior:


$$\begin{array}{c} \text{Dist\_x\_SNR}=\text{Distance}\times \text{SNR} \end{array}$$ (7)

$$\begin{array}{c} \text{RXPower\_x\_SNR}=\text{RX Power}\times \text{SNR} \end{array}$$ (8)

$$\begin{array}{c} \text{log\_Distance}=\log\left(1+\text{Distance}\right) \end{array}$$ (9)

$$\begin{array}{c} \text{log\_RXPower\_signed}=\text{sign}\left(P_{rx}\right)\cdot \log\left(1+|P_{rx}|\right) \end{array}$$ (10)

Together, these features enable the model to capture nonlinear relationships between device location, channel conditions, and the optimal spreading factor.

### 3.3 Ensemble model architecture: base learners

The proposed LSML-SF framework adopts a stacked generalization strategy in which multiple heterogeneous base learners are trained in parallel and their probabilistic outputs are combined by a meta-learner. This design is intended to leverage complementary inductive biases while reducing the risk of overfitting associated with relying on a single model. Each base learner produces a class-probability vector over the six SF classes, and these vectors are later aggregated using out-of-fold predictions to ensure unbiased meta-learning. A summary of the base learners, their key configurations, and their respective roles within the stacked ensemble is provided in Table 3. As illustrated in Figure 2, the three base learners are trained in parallel within a 3-fold cross-validation setup, and their out-of-fold probability outputs are combined by a logistic regression meta-learner.

**Table 3:** Overview of the base learners used in the LSML–SF-stacked ensemble, summarizing their main configurations and the specific contribution of each model to the overall classification framework.

**Model**	**Key configuration**	**Role in ensemble**
Linear SGD (log-loss)	Imputation and standardization; SGD optimization with log-loss	Captures global linear trends with low variance
XGBoost	600 trees; depth 6; learning rate 0.05; subsampling and regularization	Models nonlinear feature interactions
DNN	128–64 ReLU layers; dropout 0.35; focal loss (γ = 2)	Learns complex patterns and minority SF behavior
Meta-learner	Multinomial logistic regression on OOF probabilities	Combines complementary predictions

#### 3.3.1 Linear SGD classifier (log-loss)

The first base learner is a linear classifier trained using stochastic gradient descent with log-loss optimization. This corresponds to an SGD-trained multinomial logistic regression model rather than a hinge-loss SVM. The training pipeline includes mean-value imputation to handle missing entries and feature standardization to normalize feature scales prior to optimization. This model provides a fast and stable linear baseline that captures global trends in the feature space and contributes well-calibrated probability estimates to the ensemble.

#### 3.3.2 XGBoost

The second base learner is a gradient-boosted decision tree model implemented using XGBoost (Chen and Guestrin, 2016). The model is configured with 600 estimators and a maximum tree depth of 6. Histogram-based tree construction is employed to improve training efficiency, while subsampling and column sampling (0.8 each) are applied to enhance generalization. Regularization through minimum child weight and ℓ_2_ penalties further mitigates overfitting under class imbalance and noisy channel conditions. This component is primarily responsible for capturing nonlinear interactions among spatial, temporal, and signal-related features.

#### 3.3.3 Deep neural network (DNN)

The third base learner is a compact feedforward deep neural network designed with deployment constraints in mind. The network architecture comprises an input batch-normalization layer followed by two fully connected hidden layers with 128 and 64 neurons, respectively. Each hidden layer employs ReLU activation and a dropout rate of 0.35 to mitigate overfitting. A final softmax output layer produces class-probability estimates over the six SF classes.

To emphasize accurate classification of higher SFs, which have a disproportionate impact on airtime and energy consumption, the DNN is trained using a focal loss formulation (Lin et al., 2018). This loss function down-weights well-classified samples and focuses learning on harder, minority classes. The focal loss is defined as


$$\begin{array}{c} L_{\text{focal}}=-\frac{1}{N}\overset{N}{\underset{i=1}{\sum }}\alpha_{y_{i}}{\left(1-p_{i,y_{i}}\right)}^{\gamma }\log\left(p_{i,y_{i}}\right), \end{array}$$ (11)

where *p*_*i*,_*y*__*i*__ denotes the predicted probability assigned to the true class *y*_*i*_ for sample *i*, γ = 2 is the focusing parameter, and α_*y*_*i*__ is the class-dependent weight defined in Equation 2. Model optimization is performed using the Adam optimizer with early stopping to ensure stable convergence and prevent overfitting.

### 3.4 Model training

**Figure 3:**
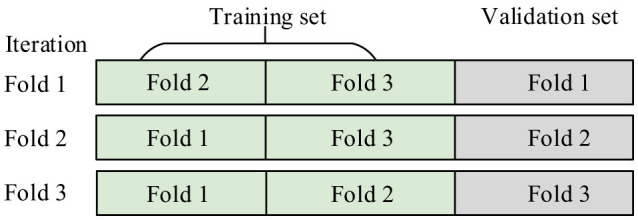
Stratified 3-Fold Cross-Validation procedure illustrating three independent training iterations, where in each iteration one fold is held out for validation and the remaining 2 folds are used for training.

A stratified 3-fold cross-validation scheme is employed to generate out-of-fold (OOF) predictions from each base learner. This procedure ensures that the meta-learner is trained exclusively on predictions from unseen data, thereby preventing information leakage. As shown in Figure 3, predictions from all base models are concatenated to form the meta-feature matrix ${\text{Z}}_{\text{OOF}}\in {\mathbb{R}}^{N\times 18}$. A multinomial logistic regression model is then trained on **Z**_OOF_ to learn the optimal combination of base learner outputs. Finally, all base models are retrained using the full dataset to construct the final ensemble.

### 3.5 Online deployment in ns-3

**Figure 4:**
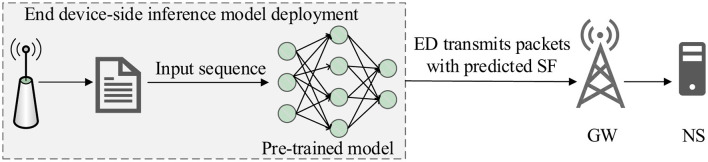
Online operation of the LSML-SF framework within the ns-3 simulator, showing the interaction between feature extraction, ensemble-based SF prediction, and adaptive packet transmission during runtime.

Figure 4 illustrates the integration of the trained LSML-SF model within the ns-3 simulation environment. When an ED initiates a packet transmission, relevant physical-layer features, including spatial coordinates, distance to the GW, *P*_*rx*_, and SNR, are collected. The engineered features described in Section 3.2 are then computed and provided as input to the pre-trained ensemble. Based on this input, the model predicts the optimal spreading factor SF^*^, which is subsequently applied to the transmission. The resulting packets are received at the GW and forwarded to the network server (NS) for standard LoRaWAN processing. This closed-loop operation enables adaptive SF selection during simulation and links offline model training with online network behavior.

## 4 Offline performance evaluation

This section evaluates the offline performance of the proposed stacked ensemble for SF classification. We report class-wise behavior through confusion-matrix analysis, examine DNN training dynamics across folds, and summarize the computational and memory requirements relevant to deployment on constrained IoT devices.

### 4.1 Confusion matrix analysis

**Figure 5:**
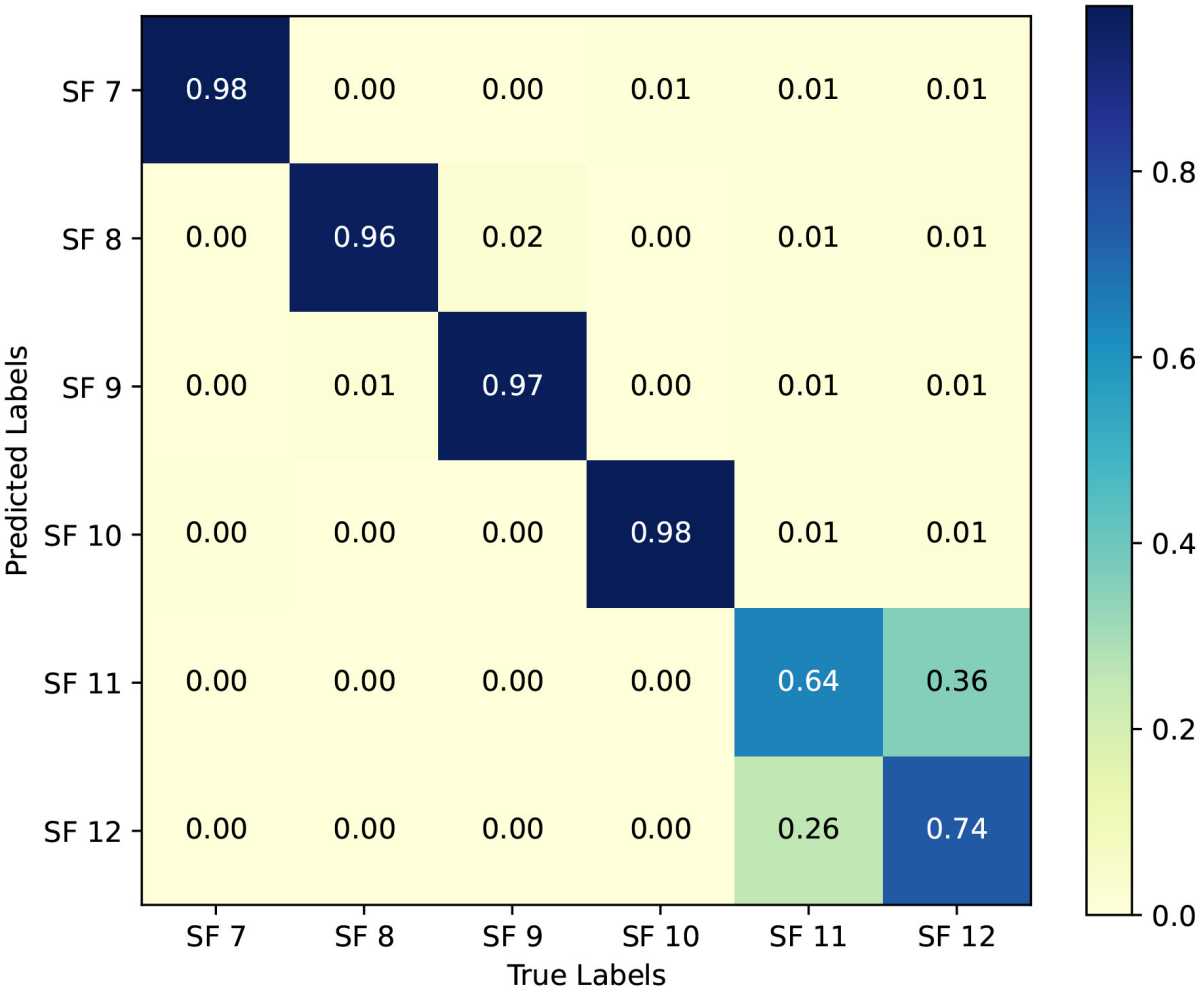
Normalized out-of-fold confusion matrix for the proposed stacked ensemble, showing per-class prediction accuracy and misclassification patterns across SF7–SF12.

The class-wise performance of the final model, obtained using stratified 3-fold cross-validation, is summarized by the normalized confusion matrix in Figure 5. Overall, the model achieves strong discrimination across SF classes, with particularly high accuracy for SF7–SF10 (above 96% on the diagonal entries).

To complement Figure 5, Table 4 reports the raw out-of-fold counts across all *N* = 225, 109 samples. For SF7–SF10, the model correctly classifies more than 36, 000 samples per class, and confusion between these lower SFs remains limited (typically below 1% of the class total). This behavior is operationally desirable because lower SFs support higher data rates and reduce airtime when link conditions permit.

**Table 4:** Raw out-of-fold confusion matrix for the stacked ensemble model across *N* = 225, 109 samples, reporting absolute classification counts for each SF.

**T\P**	**SF7**	**SF8**	**SF9**	**SF10**	**SF11**	**SF12**	**Row total**
SF7	36,710	99	58	241	214	287	37,609
SF8	79	36,110	721	89	196	305	37,500
SF9	68	556	36,265	110	204	297	37,500
SF10	177	63	96	36,663	199	302	37,500
SF11	0	0	0	0	24,126	13,374	37,500
SF12	0	0	0	0	9,568	27,932	37,500
Col. Total	37,034	36,828	37,140	37,103	34,507	42,497	225,109

Rows correspond to the true classes (T) and columns to the predicted classes (P).

A clearer separation is observed up to SF10, whereas SF11 and SF12 exhibit mutual confusion. Specifically, 13, 374 of the 37, 500 true SF11 samples are predicted as SF12 (35.7%), and 9, 568 of the 37, 500 true SF12 samples are predicted as SF11 (25.5%). This pattern is consistent with the fact that SF11 and SF12 occupy the most robust operating region and are typically triggered under similarly challenging link conditions. In practical terms, confusing SF11 and SF12 is less harmful than selecting an excessively high SF for a link that could reliably operate at SF7–SF9. Moreover, the tendency to predict the more robust SF12 for borderline cases prioritizes reliability, at the cost of modestly increased airtime.

### 4.2 Training dynamics and convergence

**Figure 6:**
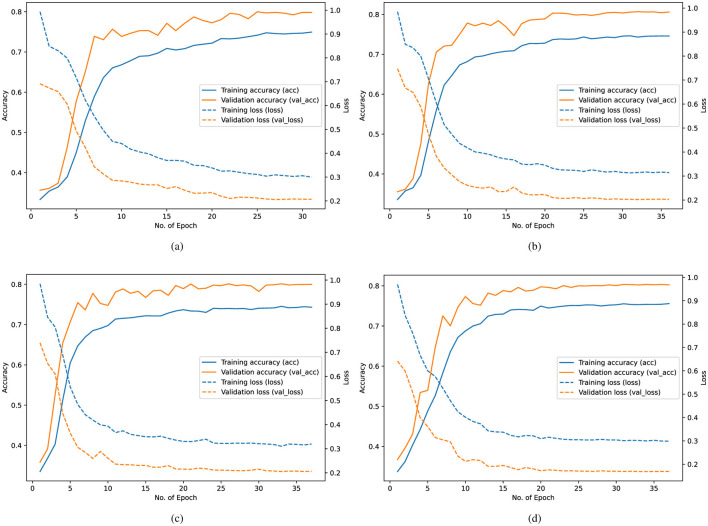
Training behavior of the DNN across the three cross-validation folds and the final refitted model. Subfigures **(a–c)** illustrate the evolution of accuracy and loss over training epochs for each fold, while **(d)** shows the corresponding curves obtained after retraining on the complete dataset.

Figure 6 reports the training/validation accuracy and loss of the DNN base learner across the three folds and the final refit model. The curves provide a direct view of convergence behavior and generalization consistency.

Across folds (Figures 6a–c), both training and validation losses decrease smoothly, and the accuracy curves remain stable without noticeable oscillations. The close tracking of validation curves relative to training curves suggests limited overfitting and indicates that the DNN component generalizes well under the chosen hyperparameters and optimization settings. Convergence typically occurs within 20–35 epochs, consistent with the configured early stopping criterion. The refit model in Figure 6d follows the same trend, supporting the stability of training when the full dataset is used.

### 4.3 Computational and memory footprint

To assess deployment feasibility on constrained EDs, we report computational complexity and memory/storage requirements. The DNN base learner is the most compute-intensive component of the ensemble and is therefore profiled in Table 5. It contains 12, 602 trainable parameters and requires 12, 288 MACs per inference pass. This footprint is compatible with embedded platforms that can sustain millions of MACs per second under typical duty-cycle constraints.

**Table 5:** Computational profile of the DNN base learner, reporting trainable parameters and multiply–accumulate operations (MACs) per forward pass.

**Layer**	**Type**	**Parameters**	**MACs**
Input	–	–	–
Batch Normalization	BatchNorm	116	0
Dense (128 units)	Dense	3,840	3,712
Dropout (0.35)	Dropout	0	0
Dense (64 units)	Dense	8,256	8,192
Dense (6 units)	Dense	390	384
Total	–	12,602	12,288

Table 6 summarizes memory and storage requirements across training and deployment. During offline training, the feature matrix for 225, 109 samples and 29 engineered features occupies approximately 52.2 MB in RAM. For deployment, the serialized ensemble totals about 20.6 MB, dominated by the XGBoost component (20.4 MB), while the DNN requires 190 KB and the linear models remain below 5 KB each. At deployment time, only the trained inference components of the ensemble are active, and the large feature matrices used during offline training are not resident in memory. Consequently, runtime memory usage is dominated by the loaded model parameters and minimal buffering required for feature computation. While this study reports serialized model sizes rather than full framework-level runtime measurements, inference is executed for each packet transmission as part of SF selection. However, given that an incorrect high-SF assignment can increase airtime and energy consumption by an order of magnitude, the measured inference complexity of approximately 12.3k MACs per decision is negligible relative to the energy savings achieved through reduced retransmissions and shorter airtime.

**Table 6:** Memory and storage footprint of LSML-SF.

**Component**	**Size**
Feature matrix (225k samples × 29 features)	52.2 MB (RAM, training)
XGBoost model (.json)	20.4 MB
DNN model (.keras)	190 KB
SVM model (.joblib)	4.1 KB
Stacker (LogReg)	1.8 KB
Feature names & Label encoder	~1 KB
Total storage for deployment	~20.6 MB

Training memory refers to the offline feature matrix; deployment storage reports serialized model sizes.

## 5 Experimental results

We next evaluate the online network impact of LSML-SF using ns-3 simulations that integrate the trained model. The analysis focuses on packet success ratio (PSR) in confirmed mode and average energy consumption per transmission, which together reflect reliability and energy efficiency under mobility and increasing network density.

### 5.1 Simulation environment

We consider EDs operating in confirmed mode under a single-GW deployment with a 5 km coverage radius. To represent industrial asset-tracking mobility, a two-dimensional random-walk model is used (Farhad et al., 2022a; GSMA-3GPP, 2016). Each ED generates six uplink packets per hour over a 24-h simulation cycle, and results are averaged over ten independent runs (Bessa et al., 2022; Ribeiro et al., 2020). The complete set of simulation parameters is provided in Table 7. The selected simulation parameters, including network size, coverage radius, mobility speed, packet generation rate, and confirmed transmission mode, were chosen to reflect commonly reported configurations in LoRaWAN performance studies and industrial IoT deployment scenarios (Nisar et al., 2025a; Farhad et al., 2025). These settings are consistent with prior simulation-based evaluations and aim to represent moderate- to high-density mobile LoRaWAN deployments under realistic operating conditions (Ali Lodhi et al., 2024; Nisar et al., 2025b; Lodhi et al., 2025).

**Table 7:** ns-3 simulation parameters used for evaluating LSML-SF under mobility in confirmed mode.

**Parameter**	**Description and value**
Maximum UL transmissions	8 (including 7 retransmissions)
ED movement speed [m/s]	1.0–2.0 (Farhad et al., 2020)
Change in direction	After 200 m
Frequency region	EU-868 MHz
UL channels	[868.1, 868.3, 868.5] MHz
Initial SF and TP	SF = 12 and TP = 14 dBm

### 5.2 Performance metrics

Two metrics are used to assess network behavior: (1) packet success ratio (PSR) in confirmed mode, reflecting end-to-end reliability; and (2) average energy consumption per transmission, reflecting energy efficiency per packet under the applied SF selection policy.

**Figure 7:**
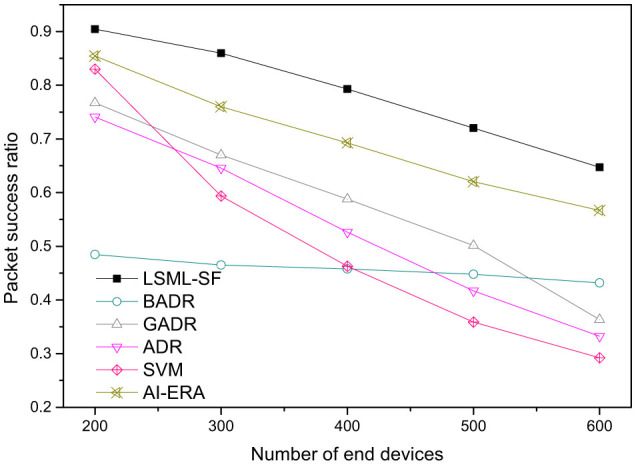
Packet Success Ratio (PSR) vs. the number of end devices under confirmed mode for LSML-SF and baseline ADR strategies.

PSR is evaluated as the number of EDs increases from *N* = 200 to *N* = 600. As shown in Figure 7, LSML-SF maintains higher PSR than ADR, BADR, GADR, SVM, and AI-ERA (Farhad and Pyun, 2023a). At *N* = 200, LSML-SF achieves PSR close to 0.9, and decreases to around 0.65 at *N* = 600 as collisions and contention increase. In contrast, ADR drops more sharply and falls below 0.3 at high density, reflecting its reactive adjustment strategy and reliance on historical link estimates (Farhad et al., 2020). The stacked ensemble combines complementary decision patterns from SGD-based classification, gradient boosting, and the DNN, resulting in SF assignments that better match link and mobility conditions. Across the tested range, LSML-SF provides a consistent PSR advantage over AI-ERA and SVM, particularly at moderate to high network loads (Farhad and Pyun, 2023b).

**Figure 8:**
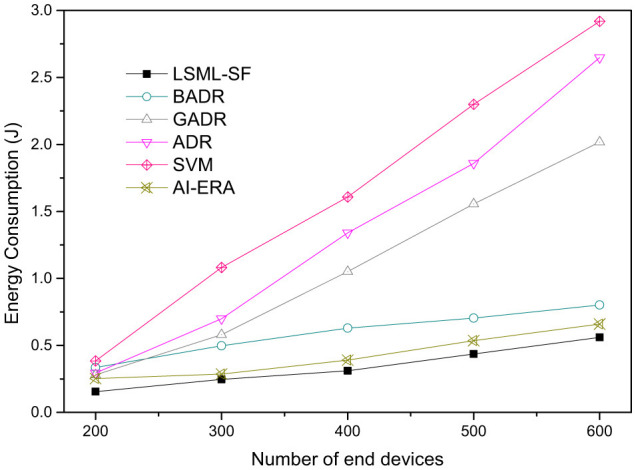
Average energy consumption per transmission (J) vs. the number of end devices for LSML-SF and baseline methods.

Figure 8 shows the average energy consumption per transmission as the network scales from 200 to 600 devices. LSML-SF maintains the lowest average energy consumption across the full range, consistent with SF selections that reduce airtime and limit retransmissions under mobility. At lower densities, the energy curves of LSML-SF, BADR, and AI-ERA remain relatively close, indicating comparable efficiency when contention is mild. As density increases, the separation becomes more visible and LSML-SF retains a lower energy profile as retransmission probability rises. As formal statistical significance testing was not conducted, these differences are reported as consistent empirical trends across simulation runs rather than statistically bounded separations. In contrast, ADR and SVM incur substantially higher energy consumption at moderate to high densities due to frequent packet losses and repeated retransmissions (Finnegan et al., 2020; Farhad et al., 2025).

### 5.3 Packet loss ratios (PLRs)

To better interpret packet delivery failures, packet loss ratios (PLRs) are decomposed into four categories:

- **PLR-I (Interference losses)**: packet collisions due to intra- or inter-SF interference that reduce SINR below the decoding threshold at the GW.
- **PLR-R (Reception-path losses)**: losses caused by limited demodulation paths at the GW when concurrent receptions exceed hardware constraints.
- **PLR-S (Sensitivity losses)**: losses occurring when received power falls below the SF-specific sensitivity threshold.
- **PLR-T (Transmission-priority losses)**: losses caused when downlink ACK scheduling pre-empts simultaneous uplink reception.

**Figure 9:**
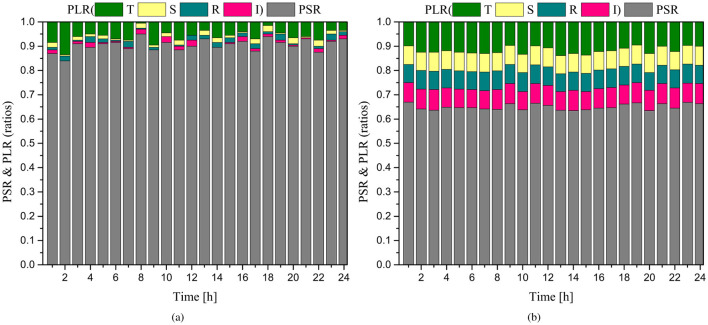
Per-hour PSR and packet loss ratios (PLRs) over 24 h under mobility for: **(a)** *N* = 200 and **(b)** *N* = 600 end devices.

Figure 9 reports per-hour PSR and the associated PLR components over 24 h for *N* = 200 (Figure 9a) and *N* = 600 (Figure 9b). For *N* = 200, PSR remains close to 90% across the day and PLR components remain small. For *N* = 600, the higher traffic load increases all PLR components, with PLR-I and PLR-S contributing most strongly, and PSR stabilizes around 65%. This highlights the combined impact of contention, sensitivity constraints, and GW resource limits as the network scales under mobility.

## 6 Limitations of the proposed study

Although the proposed LSML-SF framework demonstrates significant improvements in classification accuracy and network reliability performance, this study is subject to several limitations that provide a clear direction for future research.

**First**, the network topology used for evaluation was limited to a single-GW scenario. This setup, while common for initial validation, does not capture the complexities of large-scale, multi-GW networks. In such environments, critical factors including handovers, GW selection algorithms, and inter-GW interference could influence the performance of the SF allocation strategy.

**Second**, the proposed LSML-SF framework was implemented for on-device inference. A more energy-efficient alternative for power-constrained EDs would be to execute the ML model on the NS. Future work should integrate the pre-trained model into the NS and leverage the existing LoRaWAN MAC command control framework to downlink optimal SF assignments to the EDs, thereby shifting the computational burden away from the battery-operated devices.

**Third**, the primary validated contributions of this study lie in improved mobility robustness and energy efficiency, while interpretability is identified as an important direction for future research in ML-driven LoRaWAN resource allocation. The evaluation presented in this study is based exclusively on ns-3 simulations, which, while widely adopted in LoRaWAN research, cannot fully capture real-world factors such as hardware variability, uncontrolled interference sources, and deployment-specific environmental conditions. Validation of the proposed framework using real-world testbeds and heterogeneous hardware platforms is, therefore, identified as an important direction for future work.

## 7 Conclusions

This study presented LSML-SF, a lightweight stacked-ensemble for mobility-aware SF allocation in LoRaWAN. The approach combines a linear classifier, gradient boosting, and a compact DNN through stratified out-of-fold stacking, trained on a simulator-derived dataset enhanced with 29 engineered features. Offline, the model achieved approximately 85% out-of-fold accuracy; online, when integrated into ns-3, it consistently improved packet success ratio (PSR) and reduced energy per transmission across 200–600 mobile end devices compared to ADR, BADR, and representative ML baselines. Hourly analysis further decomposed reliability outcomes via packet loss ratios (PLR-I, PLR-R, PLR-S, and PLR-T), clarifying how the method mitigates interference, sensitivity limitations, demodulator contention, and ACK-priority effects under mobility.

A compute and memory audit indicated the feasibility of the learned components for constrained deployments: The DNN base learner requires roughly 12.6k parameters and about 12.3k MACs per inference, while the end-to-end pipeline can be realized in ns-3 by constructing online input sequences from the same feature family used offline. These attributes position LSML-SF as a practical path toward reliability- and energy-aware SF control in dynamic environments.

Future work will (i) extend evaluation to multi-GW topologies with handover and duty-cycle constraints, (ii) broaden the baselines to include recent ADR variants and reinforcement learning methods with statistical significance tests, (iii) provide ablations on feature groups and model components, and (iv) quantify end-to-end latency and memory for the full ensemble under strict MCU budgets or migrate inference to the network server while preserving responsiveness. Together, these steps will further mature LSML-SF from a simulator-validated approach to a field-ready solution for mobile IoT networks.

## Data Availability

The datasets presented in this study can be found in online repositories. The names of the repository/repositories and accession number(s) can be found below: https://github.com/afarhad/AI-ERA/tree/main.

## Footnotes

1
https://github.com/afarhad/AI-ERA/tree/main/data
