# Correction: LSML-SF: a lightweight stacked ML approach for spreading factor allocation in mobile IoT LoRaWAN networks

**DOI:** 10.3389/frai.2026.1819135

**Published:** 2026-03-30

**Authors:** Arshad Farhad, Muhammad Ali Lodhi, Farhan Nisar, Hassan Jalil Hadi, Naveed Ahmad, Mohamad Ladan

**Affiliations:** 1 Department of Computer Science, Bahria University, Islamabad, Pakistan; 2 School of Information Engineering, Yangzhou University, Yangzhou, China; 3 Department of Physical and Numerical Science Qurtaba University & IT, Peshawar, Pakistan; 4 Center of Excellence in Cyber Security, Prince Sultan University, Riyadh, Saudi Arabia; 5 Software Engineering Department, Prince Sultan University, Riyadh, Saudi Arabia; 6 College of Computer and Information Sciences, Prince Sultan University, Riyadh, Saudi Arabia

**Keywords:** Internet of Things (IoT), LoRaWAN, machine learning, resource allocation, spreading factor, stacked generalization

The funder Prince Sultan University was erroneously omitted.

The original statement read:

“The author(s) declared that financial support was not received for this work and/or its publication.”

The correct **Funding** statement is:

The author(s) declared that financial support was received for this work and/or its publication. The authors would like to thank Prince Sultan University for paying the Article Processing Charges (APC) of this publication.

The original version of this article has been updated.

